# ISL1 promotes enzalutamide resistance in castration-resistant prostate cancer (CRPC) through epithelial to mesenchymal transition (EMT)

**DOI:** 10.1038/s41598-021-01003-0

**Published:** 2021-11-09

**Authors:** Jae Duck Choi, Tae Jin Kim, Byong Chang Jeong, Hwang Gyun Jeon, Seong Soo Jeon, Min Yong Kang, Seon Yong Yeom, Seong Il Seo

**Affiliations:** 1 Department of Urology, Nowon Eulji Medical Center, Eulji University School of Medicine, Seoul, Republic of Korea; 2 Division of Immunology, Department of Molecular Cell Biology and Samsung Biomedical Research Institute, Sungkyunkwan University School of Medicine, Suwon, Republic of Korea; 3 Department of Urology, Samsung Medical Center, Sungkyunkwan University School of Medicine, Seoul, Republic of Korea

**Keywords:** Cancer, Genetics, Immunology, Molecular biology, Oncology, Urology

## Abstract

Abnormal expression of insulin gene enhancer-binding protein 1 (ISL1) has been demonstrated to be closely associated with cancer development and progression in several cancers. However, little is known about ISL1 expression in metastatic castration-resistant prostate cancer (CRPC). ISL1 has also been recognized as a positive modulator of epithelial–mesenchymal transition (EMT). In this study, we focused on ISL1 which showed maximum upregulation at the mRNA level in the enzalutamide-resistant cell line. Accordingly, we found that ISL1 was overexpressed in enzalutamide-resistant C4-2B cells and its expression was significantly related to EMT. Our findings reveal the important role of ISL1 in androgen receptor (AR)-dependent prostate cancer cell growth; ISL1 knockdown reduced the AR activity and cell growth. ISL1 knockdown using small-interfering RNA inhibited AR, PSA, and EMT-related protein expression in C4-2B ENZR cells. In addition, knock-down ISL1 reduced the levels of AKT and p65 phosphorylation in C4-2B ENZR cells and these suggest that knock-down ISL1 suppresses EMT in part by targeting the AKT/NF-κB pathway. Further, ISL1 downregulation could effectively inhibit tumor growth in a human CRPC xenograft model. Together, the present study shows that downregulation of ISL1 expression is necessary for overcoming enzalutamide resistance and improving the survival of CRPC patients.

## Introduction

Androgen deprivation therapy (ADT) is the mainstay treatment for advanced prostate cancer (PCa), given the importance of androgens for PCa development. Despite the initial response to ADT, the disease typically progresses to a castration-resistant state, i.e., castration-resistant prostate cancer (CRPC)^1,2^. Many studies show that androgen receptor (AR) signaling still plays a critical role in CRPC^3^. The use of AR inhibitor enzalutamide for the treatment of CRPC has been recently approved by the Food and Drug Administration^4^. However, the development of enzalutamide resistance has already been reported in a majority of CRPC patients. Known resistance mechanisms include de novo androgen biosynthesis, expression of AR splice variants, Wnt/β-catenin pathway activation, and cholesterol biosynthesis^5–8^. The identification of critical molecular and cellular events associated with tumor progression, invasion, and metastasis to the bone as well as other sites has provided new insights with respect to targeting advanced disease^9^. Epithelial–mesenchymal transition (EMT) is a process by which epithelial cells undergo morphological changes to acquire a motile mesenchymal phenotype. This phenomenon is implicated not only in cancer metastasis but also in the development of therapeutic resistance^10,11^. Hence, the targeting of EMT can serve as a new potential strategy for the treatment of CRPC through the reversion of the invasive mesenchymal phenotype to a well-differentiated tumor epithelial tumor phenotype.

Insulin gene enhancer-binding protein 1 (ISL1), a LIM homeodomain transcription factor, plays an important role in the development of pancreatic islets of Langerhans during embryogenesis. ISL1-deficient mouse embryos fail to exhibit heart development and the differentiation of the neural tube into motor neurons^12–14^. The association between aberrant ISL1 expression and cancer progression is being gradually recognized. For instance, abnormal expression of ISL1 has been demonstrated to be closely associated with cell proliferation and invasion in several cancers^15,16^. In addition, ISL1 has been known to serve as a novel regulator of cyclin D1, cyclin B, and c-myc genes in cancer^17,18^.

ISL1 may act as a positive modulator of EMT, a critical regulator of cancer stem cell (CSC) phenotype. This is especially important as CSCs are a subpopulation of neoplastic cells with stem cell-like properties such as the ability to self-renew and undergo metastasis^19–21^. EMT-inducing transcription factors (EMT-TFs) can be typically classified into three different protein families, namely, the Snail, ZEB1, and basic helix-loop-helix families^22^. The contribution of EMT to the CSC phenotype is thought to be dependent on the cell type and/or coexisting genetic/epigenetic abnormalities, and abnormal EMT and epigenetic changes are known to be related to cancer metastasis and tumor relapse^21^. Here, we demonstrate that ISL1 is overexpressed in enzalutamide-resistant C4-2B cells and that its expression is significantly related to EMT. Targeting ISL1 expression with a small-interfering RNA (siRNA) resulted in the inhibition of the proliferative and invasive capabilities of enzalutamide-resistant C4-2B cells and colonization abilities of enzalutamide-resistant C4-2B cells in a mouse xenograft model. However, the molecular basis underlying these effects is not well understood, and to the best of our knowledge, has not been investigated in the context of PCa.

## Methods

### Materials and methods

#### Cell culture and reagents

LNCaP and C4-2B cells were purchased from the American Type Culture Collection (Rockville, MD, USA). The C4-2B MDVR cell line was generated by culturing C4-2B cells in a medium supplemented with 20 µM enzalutamide. Cells were cultured in RPMI-1640 containing 10% FBS. Tumor spheres were cultured in serum-free DMEM/F12 supplemented with bFGF (20 ng/mL; Invitrogen), EGF (20 ng/mL; BD Biosciences), and N2 supplement (1 ×; Invitrogen). Enzalutamide was obtained from Selleckchem, and dihydrotestosterone (DHT) from Sigma. Cells were transfected with pCMV6-DDK (vector) or pCMV6-ISL1 using TurboFectin reagent (OriGene Technologies Inc, Rockville, MD, USA). The detailed procedures of siRNA transfection were described previously^16^. siRNA against human ISL1 (cat#L-011707-00-0005), and non-targeting control siRNA (cat#D-001810-10-20) were purchased from GE Dharmacon.

#### RNA isolation and reverse-transcription quantitative polymerase chain reaction (RT-qPCR)

Total RNA was extracted using RNeasy Purification Kit (Qiagen), and qRT-PCR was carried out with TaqMan primer and probe sets (Applied Biosystems). Data were normalized to GAPDH. *ISL1*, *KLK2*, *KLK3*, *TMRPSS2*, and *IGF1R* primers and probes were designed as per Applied Biosystems (Assay IDs: Hs00158126_m1, Hs00428383_m1, Hs02576345_m1, Hs00237175_m1, Hs00609566_m1).

#### RNA sequencing

RNA sequencing data was extracted from microarray gene expression previously performed^5,23,24^.

#### Colony formation assay

For the colony formation assay, 1000 cells were seeded in six-well plates. Cells were cultured for 21 days and stained with 0.1% crystal violet. The cell colonies were imaged and the dye was subsequently extracted using 10% acetic acid. The absorbance was determined by spectrophotometry (570 nm).

#### Screening for RTK expression in cells

Cell lysates were collected using lysis buffer and incubated with Phospho-Receptor Tyrosine Kinase Array membranes (R&D Systems), according to the manufacturer’s instructions.

#### Cell growth and proliferation assessment

To assess the cell number, an equal volume of 0.4% (w/v) trypan blue was added to each cell suspension, and cell viability was determined based on the ability of live cells to exclude trypan blue. Viable cells were counted using a hemocytometer. Cell proliferation was quantified using CCK-8 assay (Dojindo) as per manufacturer’s protocol.

#### Western blot analysis

Cells were lysed in buffer containing 150 mM NaCl, 0.5% NP-40, 0.5% sodium deoxycholate, 0.1% sodium dodecyl sulfate (SDS), 50 mM Tris, pH 8.0, and a protease inhibitor cocktail (Roche Applied Science, Vienna, Austria). Cell lysates were separated on sodium dodecyl sulfate (SDS) polyacrylamide gels and the separated protein bands were transferred onto an Immobilon-P membrane (Millipore, Darmstadt, Germany). The membrane was blocked with a solution containing 5% skim milk and 0.1% Tween-20 for 1 h and then probed overnight with the indicated primary antibodies at 4 °C. The membrane was then probed with a horseradish peroxidase-conjugated secondary antibody (1:2000; Cell Signaling Technology Inc, Danvers, MA, USA) for 1 h and developed using the ECL-Plus Kit (Thermo Scientific, Rockford, IL). Antibodies against ISL1, β-actin (both Santa Cruz Biotechnology), E-cadherin, N-cadherin (both BD Biosciences), phospho-AKT, phosphor-p65, p65, AKT, Snail, AR, and PSA (Cell Signaling Technology) were used as the primary antibodies. The membrane was cut prior to hybridization with antibodies. Uncropped images of western blotting used in this article can be found in Supplementary Figures as indicated in each figure legend.

#### Transwell migration assay

The detailed procedures were described previously^16^.

#### Evaluation of the antitumor potential of ISL1 in vivo

Six-week-old female BALB/C nu/nu mice were subcutaneously injected with 1 × 10^7^ human C4-2B cells—expressing the indicated short-hairpin RNA (shRNA)—in their right flanks. For the injection, cells were suspended in 100 μL of 50% Matrigel (BD, NJ) in complete media. The body weight of mice was measured, and tumor growth was monitored using Vernier calipers for up to 24 days after cell injection. The tumor volume was calculated as follows: tumor volume (mm^3^) = length × (width)^2^ × 0.5. At necropsy, the tumors were dissected and weighed. After sacrifice, tumors were harvested.

#### Statistical analysis

Data in the graphs represent mean ± standard deviation (SD) of values from at least three independent measurements. To determine the differences in mean values, a Student’s *t* test was employed. Intergroup comparisons were performed using the paired two-sample *t* test. Differences were considered significant at *P* < 0.05.

## Results

### Identification of ISL1 in enzalutamide-resistant PCa cells

**Figure 1:**
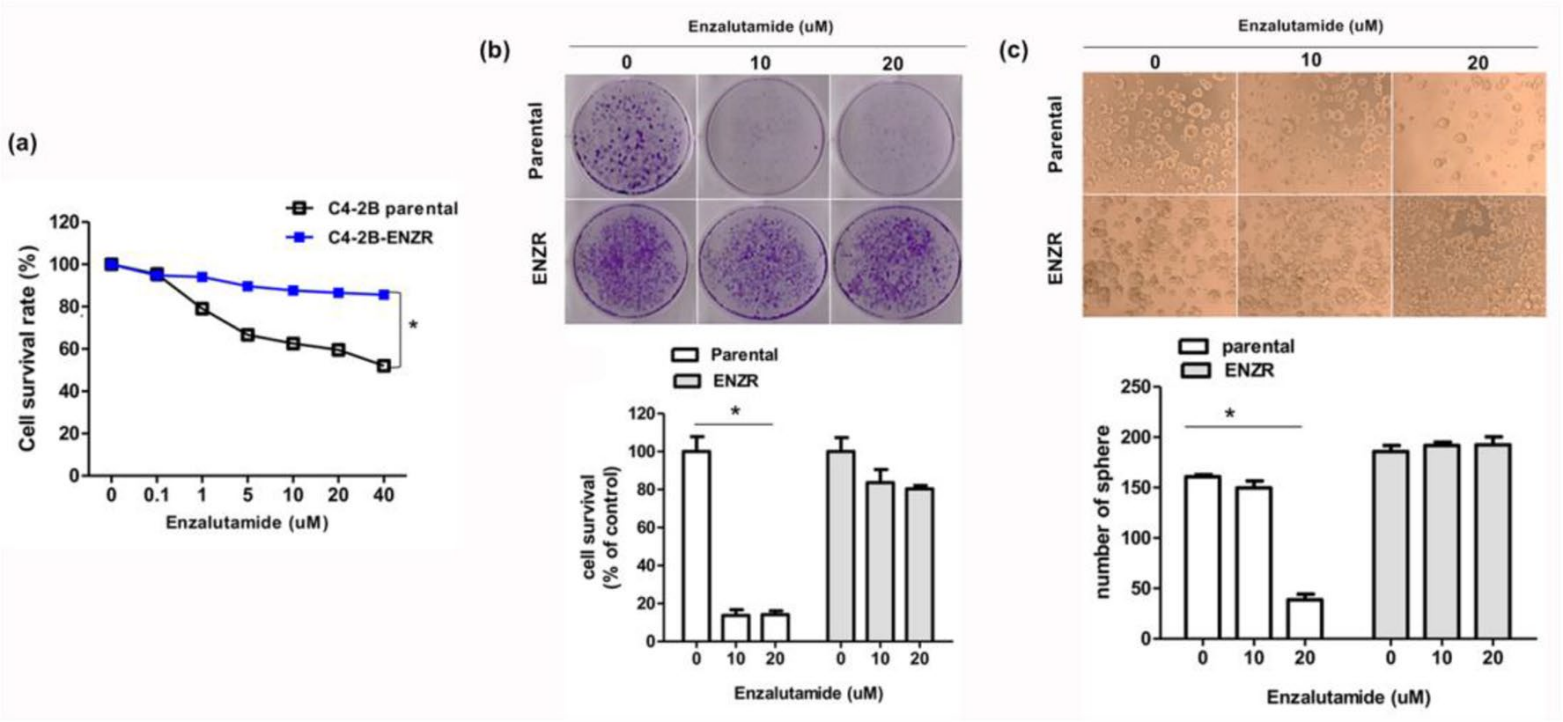
C4-2B MDVR cells are resistant to enzalutamide in vitro. (**a**) C4-2B parental and C4-2B ENZR cells were treated with different concentrations of enzalutamide for 72 h, the cells were measured using the CCK8 solution. *p < 0.005. (**b**) Crystal violet staining for colonies from the same number cells treated with the indicated concentration of enzalutamide. (**c**) C4-2B parental and C4-2B ENZR cells were seeded in an ultra-low attachment 24-well plate. (**d**) The average relative number of crystal violet staining colonies is shown in the lower panel. (**e**) The average relative number of spheroid is shown in the lower panel. Spheroid number was evaluated at 6 days post-seeding. *p < 0.005.

**Figure 2:**
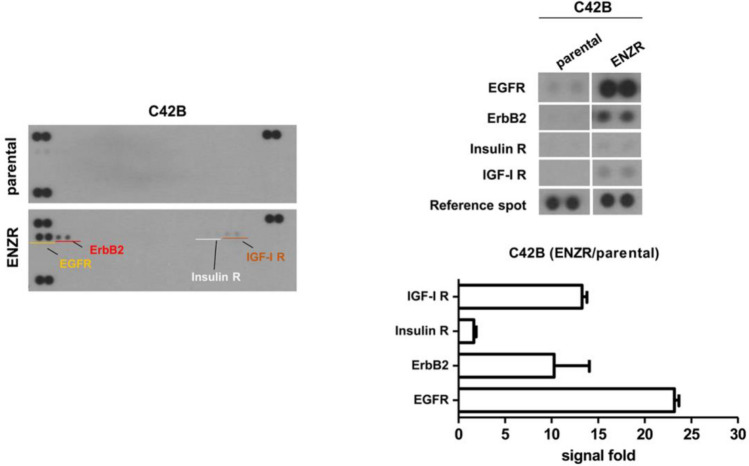
In vitro screening of enzalutamide resistant targets using RTK array analysis. (**a**) Representative blots of human receptor tyrosine kinase antibody array probed with protein lysates isolated from C4-2B cells carrying parental (upper) and ENZR (lower). (**b**) Individual RTKs are spotted and identification of phospho-EGFR, p-ErbB2, p-Insulin R and p-IGF-IR are indicated. Positive control spots are located at the corners of the human phospho-RTK array. (**c**) Spot intensity was quantified by densitometry and the data are presented in histogram format.

We established an in vitro model of enzalutamide-resistant PCa, i.e., C4-2B ENZR by culturing C4-2B cells in a medium supplemented with enzalutamide. C4-2B cells have a nearly identical AR status but show higher expression of AR variants^25,26^. To confirm enzalutamide resistance, we performed cell viability assay after treating the enzalutamide-resistant cell line with different concentrations of the drug (0.1–40 μM). As shown in Fig. 1a,b, enzalutamide significantly inhibited the proliferation and clonogenic ability of C4-2B parental cells but had little effect on C4-2B ENZR cells. As the tumor sphere formation is based on the unique property of stem/progenitor cells to survive and grow in a serum-free medium, we performed a tumor sphere formation assay to examine whether enzalutamide resistance enhances the self-renewal of PCa cells. Our data show that enzalutamide resistance enhanced the sphere formation ability of cells in a concentration-dependent manner (Fig. 1c). Furthermore, we performed a phospho-RTK activity array assay (including 42 RTKs) to identify additional RTKs that may be activated in CRPC. As shown in Fig. 2, we observed a substantial increase in the phosphorylation of ErbB family members (epidermal growth factor receptor [EGFR] and ErbB2), insulin R, and IGF-1R in C4-2B ENZR as compared to those in C4-2B parental cells. EGFR phosphorylation appeared to be the strongest among all kinases.

**Figure 3:**
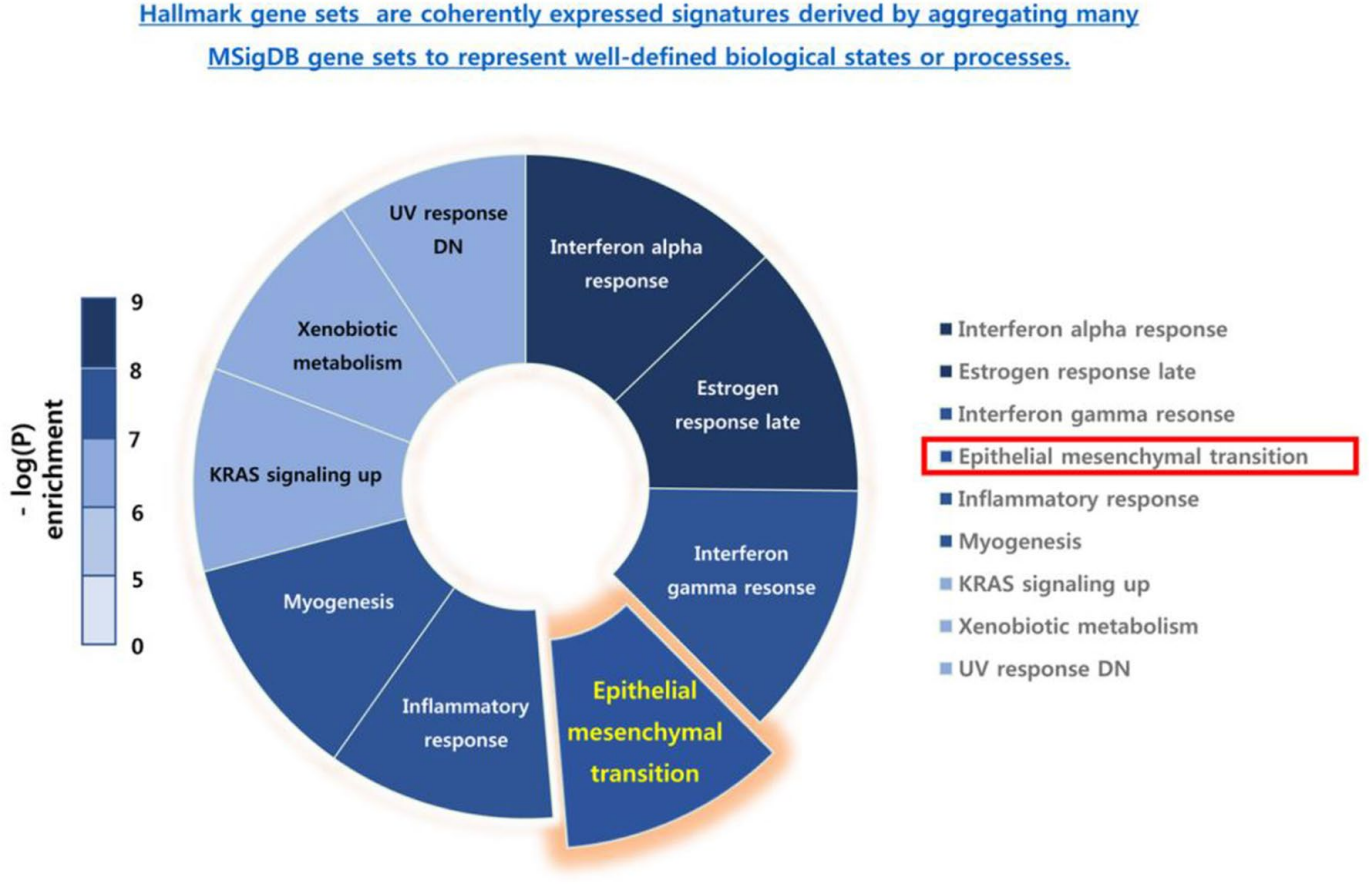
Hallmark wheels of genomic/transcriptomic and full integrated datasets, related to Fig. 4. Hallmark wheel showed enrichment of hallmark pathways when the transcriptional and genomic information is included.

**Figure 4:**
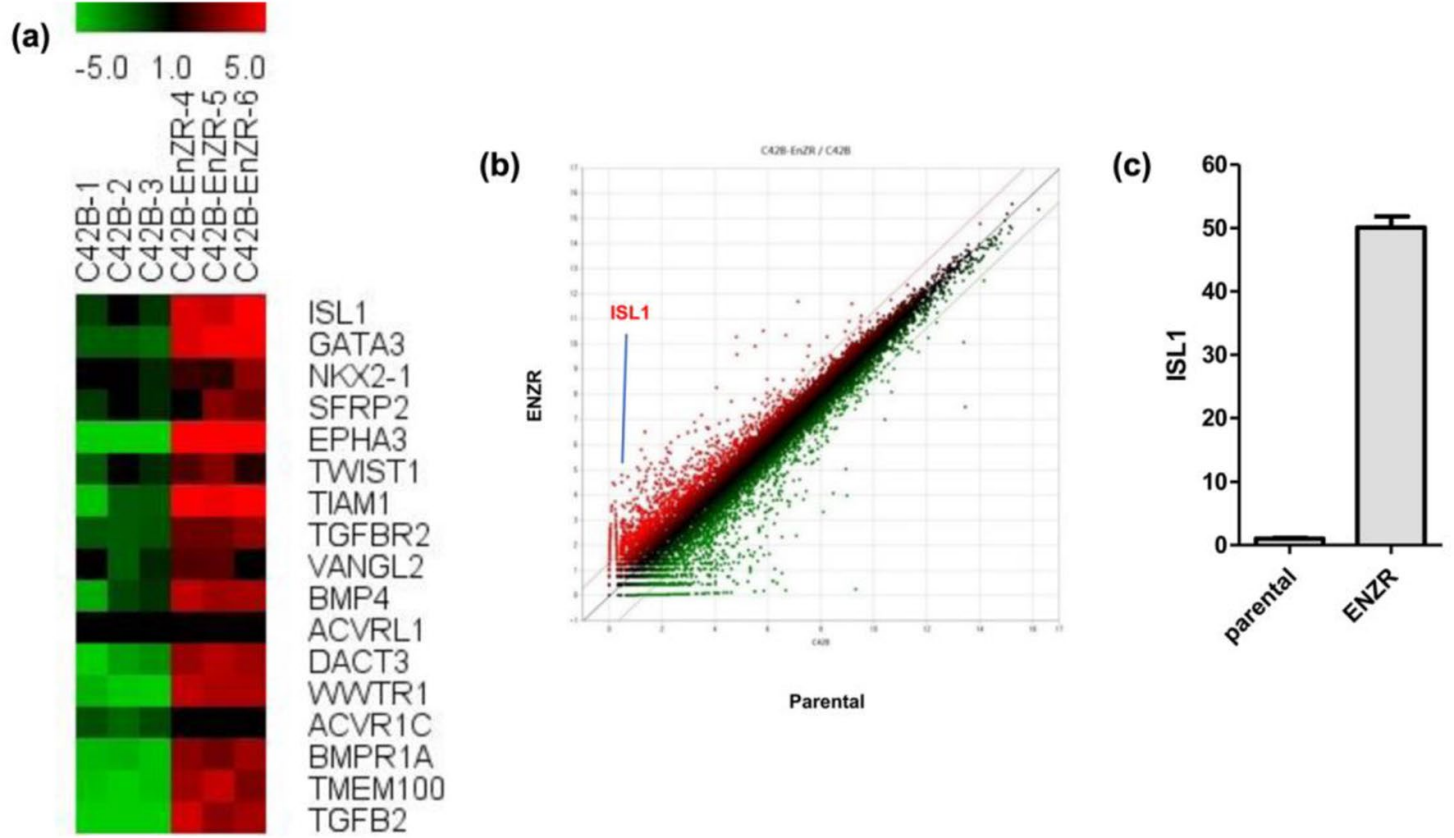
ISL1 is upregulated in enzalutamide-resistant (ENZR) prostate cancer. (**a**) Heat map of the most significantly differentially expressed epithelial mesenchymal transition genes from three independent experiments using the parental and enzalutamide-resistant C4-2B cell lines. Expression of transcripts encoding genes involved in EMT (epithelial mesenchymal transition) was analyzed by GSEA. Genes that were regulated twofold between C4-2B parental and enzalutamide-resistant C4-2B cells were enriched and heatmap was generated by TM4 Multi Experiment Viewer (MeV). GSEA, Gene Set Enrichment Analysis. (**b**) Scatterplot of transcript expression from the parental and enzalutamide-resistant C4-2B cell lines, with the position of ISL1 indicated. (**c**) The mRNA levels of ISL1 from the parental and enzalutamide-resistant C4-2B cell lines calculated from three different samples by RT-qPCR. Errors bars represent the mean ± SD of three independent experiments and * denotes *p* < 0.005 vs. the parental cell line.

After validation of the enzalutamide resistance, we analyzed global changes in the mRNA expression using quantitative mRNA-sequencing. To identify common pathways underlying the development of enzalutamide resistance, we used the complete transcriptional profile for gene set enrichment analyses (GSEA: http://www.broadinstitute.org/GSEA) and investigated the predefined oncogenic signatures and hallmarks based on analysis on the Molecular Signature Database (MsigDB). Comparison of the parental and enzalutamide-resistant C4-2B cell lines revealed changes in the expression of several major biological pathways and some EMT-related pathways. Enriched hallmark gene sets included those involved in EMT, supporting the hypothesis that resistant cells bypass EMT (Fig. 3). We focused on ISL1, which showed maximum upregulation at the mRNA level in the enzalutamide-resistant cell line as compared to that in the parental C4-2B cell line (Fig. 4a). The position of ISL1 on the scatter plot of the two different cell lines has been indicated in Fig. 4b. The change in ISL1 mRNA expression was confirmed by RT-PCR (Fig. 4c).

### ISL1 is essential for AR activity and AR-dependent cell proliferation

**Figure 5:**
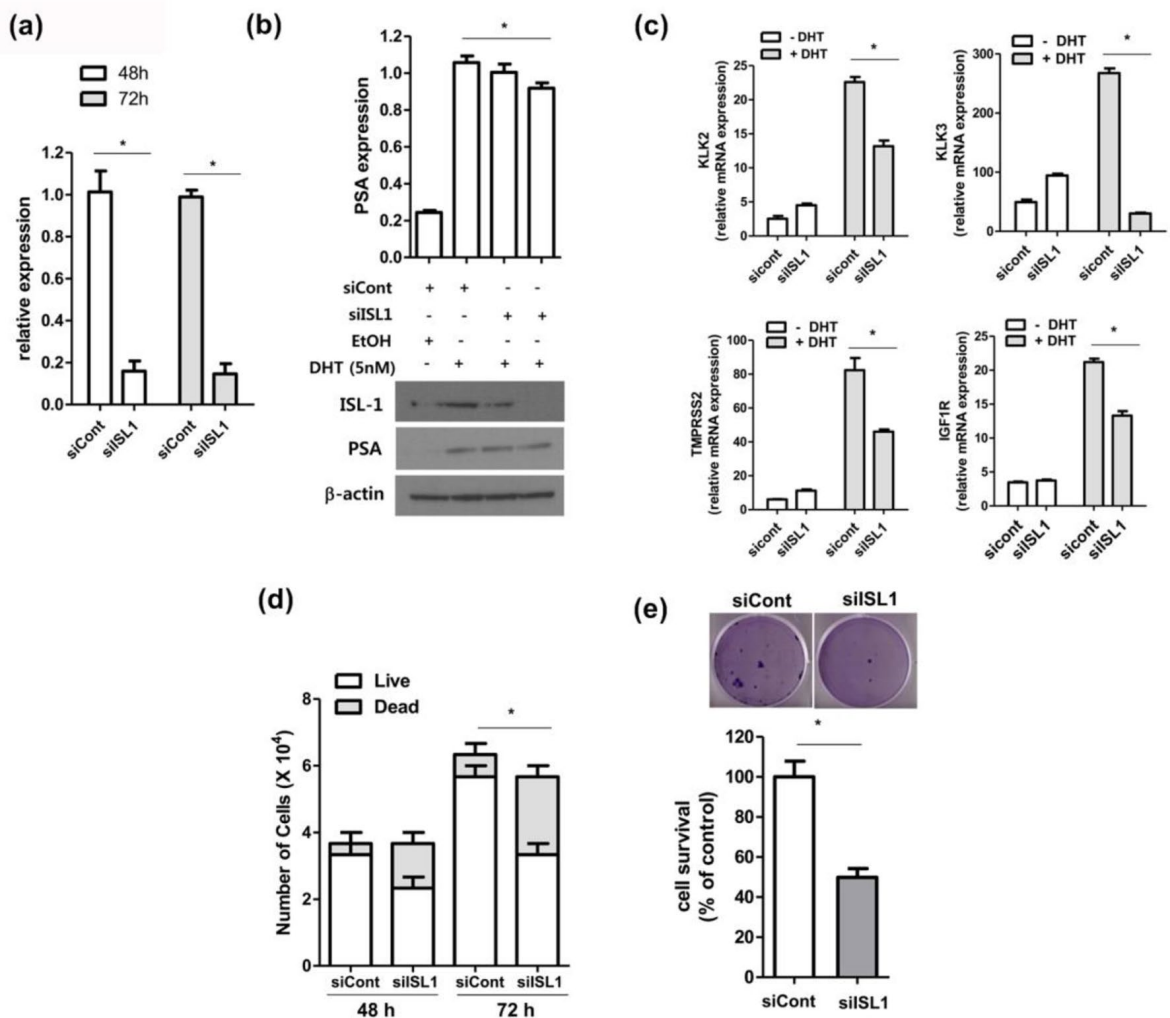
ISL1 is required for AR activity and AR-dependent cell proliferation. (**a**) The efficiency of ISL1 knock-down was measured by comparing mRNA levels of ISL1 in siRNA expressing LNCaP cell lines. ISL1 mRNA levels were measured by qRT-PCR. Error bars represent the mean ± SD of three independent experiments and * denotes *p* < 0.05 vs. the control siRNA (siCont) group. (**b**) Whole cell extracts were analyzed with anti-ISL1, anti-PSA and anti-β-actin antibodies. For the induction of AR activity, 5 nM DHT (dihydrotestosterone) was added after one day of serum deprivation. Bottom, a Western blotting experiment; top, bands on the Western blot analysis were quantified by densitometry and the data are presented in histogram format. *p < 0.05 (vs. siCont treated DHT). Full-length blots are presented in Supplementary Fig. S6. (**c**) Relative mRNA expression levels of the indicated genes were measured in ISL1 knock-down LNCaP cell lines. Error bars represent mean ± SD of three independent experiments and * denotes *p* < 0.05 vs. the control siRNA (siCont) group. (**d**) Cell viability in siISL1 transfected LNCaP cells. The cells were plated at a density of 1 × 10^4^ cells in 6 well plate for the period indicated. The detached living and dead cells were collected, stained with trypan blue and counted under a microscope using a hemocytometer. Error bars represent the mean ± SD of three independent experiments and * denotes *p* < 0.05 vs. the control siRNA dead cell (siCont) group. (**e**) Crystal violet staining for colonies from the same number of indicated siRNA expression LNCaP cells. The average relative number of colonies is shown in the lower panel. Error bars represent the mean ± SD of three independent experiments and * denotes *p* < 0.05 vs. the control siRNA (siCont) group.

To test the possible role of ISL1 in AR function in PCa cells, we used siRNA to knockdown ISL1 expression in LNCaP cells, which are known to exhibit AR activity. The knockdown efficiency of ISL1 siRNA was confirmed by RT-qPCR (Fig. 5a) and western blotting (Fig. 5b). Cells expressing the control siRNA and ISL1 siRNA were stimulated with DHT for 24 h, and the expression of AR-target genes was analyzed by western blotting (Fig. 5b) and qRT-PCR (Fig. 5c). The mRNA expression of AR target genes, including KLK3 (PSA), KLK2, TMPRSS2, and IGF1R, increased after DHT stimulation in control siRNA cells; however, ISL1 knockdown prevented the increase in target gene expression. It is well known that AR plays an important role in the growth of PCa cells. To test the possibility that ISL1 may augment AR-mediated PCa cell growth, we measured cell viability at 48 and 72 h using the trypan blue exclusion assay (Fig. 5d). The growth was slower in ISL1 knockdown cells than that in control cells under normal conditions. Thus, ISL1 may play an important role in AR-dependent PCa cell growth. We also tested the colony formation abilities of control and ISL1-knockdown cells. The number of colonies was counted, and the relative numbers were plotted (Fig. 5e). As observed with LNCaP cells, the knockdown of ISL1 expression resulted in a decrease in AR activity and cell growth.

### ISL1 depletion suppresses EMT via the AKT/nuclear factor kappa B (NF-κB) signaling

**Figure 6:**
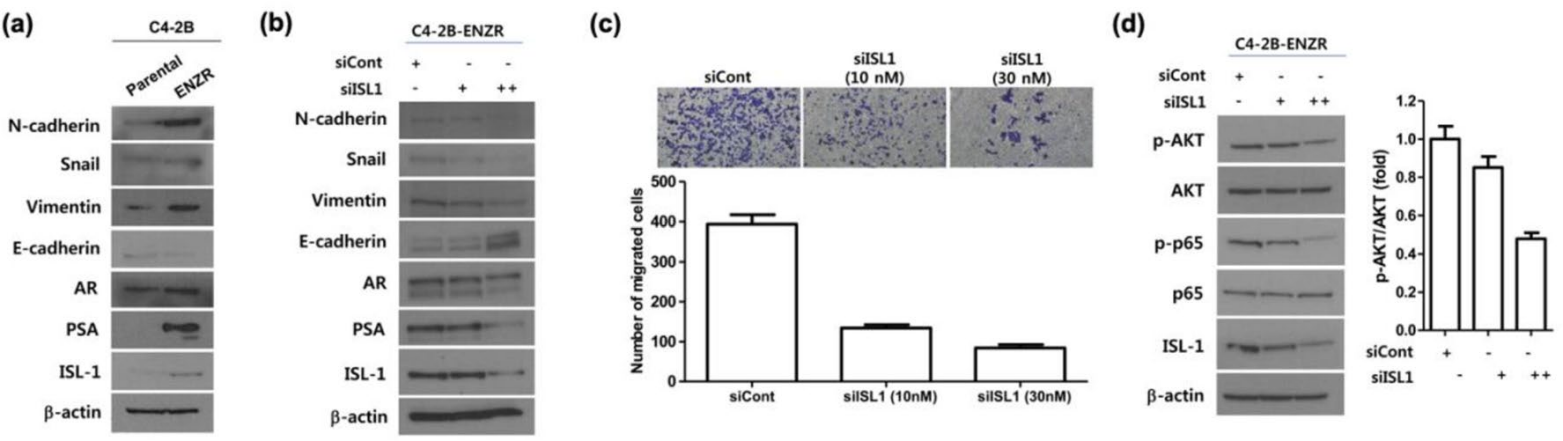
Depletion of ISL1 sensitizes enzalutamide-resistant C4-2B cells and decreases their epithelial-to-mesenchymal transition (EMT) properties. (**a**) Parental C4-2B and C4-2B-ENZR cells were subjected to western blot analysis for EMT gene expression. β-Actin was used to show equal loading. Full-length blots are presented in Supplementary Fig. S7. (**b**) C4-2B-ENZR cells transfected with siCont or siISL1 and subjected to western blot analysis for the indicated proteins. β-Actin was used to show equal loading. Full-length blots are presented in Supplementary Fig. S7. (**c**) Assessment of invasion of C4-2B-ENZR cells using Transwell migration assay. (**d**) C4-2B-ENZR cells transfected with siCont or siISL1 and the effects on p-p65 (S536) and p-AKT (S473) level were analyzed by western blot analysis; densitometry analysis of immune-reactive bands detected by western blots show that the ISL1 knockdown induced decrease in AKT phosphorylation over the basal was about twofold in C4-2B cells. Full-length blots are presented in Supplementary Fig. S7.

We determined whether ENZR cells underwent a partial EMT by performing western blotting to evaluate the expression of the markers related to either an epithelial or mesenchymal cell state (Fig. 6a). C4-2B ENZR cells showed an increase in the expression of Snail and vimentin, a loss of E-cadherin, and a gain of N-cadherin expression as compared to control cells. AR and PSA levels were upregulated in C4-2B ENZR cells as compared to those in control cells, suggesting that AR is involved in the development of enzalutamide resistance. Based on our observation that EMT-related proteins and AR are upregulated in enzalutamide-resistant cells, we assessed whether ISL1 plays a role in the induction of AR in ENZR cells and investigated the impact of ISL1 knockdown on enzalutamide resistance. ISL1 knockdown with siRNA resulted in the inhibition of AR, PSA, and EMT-related protein expression in C4-2B ENZR cells (Fig. 6b). Furthermore, the results of transwell migration assay revealed fewer migrating cells in the siISL1 group than those in the siCont group (Fig. 6c). Densitometric analysis of immune-reactive bands in western blotting revealed that ISL1 knockdown reduced p65 phosphorylation (Fig. 6d), which is essential for the nuclear translocation of NF-κB/p65. To clarify the mechanism underlying the inhibition of NF-κB signaling by ISL1, AKT expression and phosphorylation were evaluated using western blotting. Knock-down ISL1 reduced the levels of p-AKT in C4-2B ENZR cells (Fig. 6d). These results suggest that knock-down ISL1 suppresses EMT in part by targeting the AKT/NF-κB pathway. In addition, we performed gain-of-function experiments in C4-2B cells. The results showed that ISL1 (DDK-tagged full length) overexpression promoted the proliferative abilities of cells after culturing 72 h as well as enhanced the AR and EMT signaling in C4-2B cells (Supplementary Fig. S1).

### Inhibition of ISL1 helps overcome enzalutamide resistance in vivo

**Figure 7:**
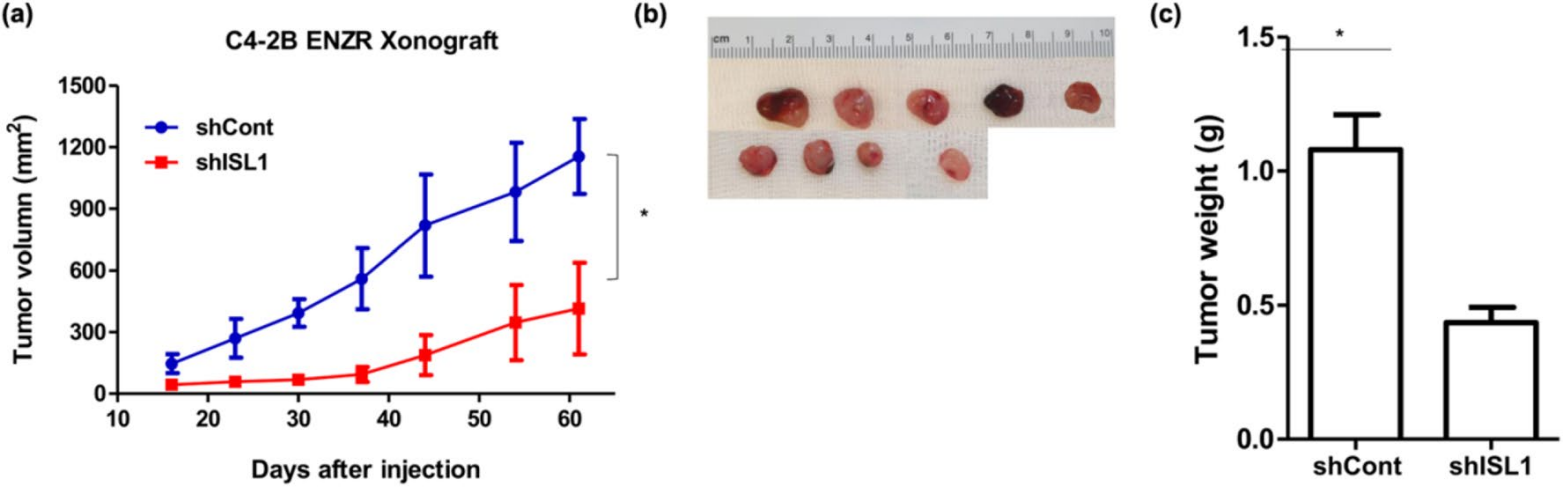
ISL1 knock-down attenuates tumor growth in enzalutamide-resistant (ENZR) prostate cancer cells. (**a**) The efficiency of ISL1 knock-down was measured by western blot analysis. b-action was used to show equal loading. (**b**) Photographs showing tumor formation in nude mice injected with stable cell lines with ISL1 knockdown. Tumor growth curves in relation to the ISL1 suppression in human CRPC C4-2B ENZR xenografts. Tumor size was measured once per week and compared between groups by ANOVA (*p < 0.001). (**c**) Relationship between tumor weights and ISL1 expression status in human CRPC xenograft model. Tumors were excised and examined at the end of the experiments. Representative graph is shown as vertical box plots and tumor weights are presented as mean (g) SD.

To validate the oncogenic effect of ISL1 in vivo, we established a human CRPC xenograft model by injecting C4-2B cells expressing either control or ISL1 shRNA in nude mice. The shRNA-mediated stable knockdown of ISL1 was confirmed by immunoblotting (Fig. 7a). We compared the increase in tumor volume over 30 days and found that the xenografts from ISL1 knockdown cells showed hardly any increase in size, whereas control shRNA-expressing cells exhibited continuous tumor growth (Fig. 7b). Tumors originating from ISL1 knockdown cells were significantly lower in weight than those from control cells (Fig. 7c). Our results demonstrate that ISL1 downregulation can effectively inhibit tumor growth in a xenograft model of human CRPC. Not only expression of AR and EMT-related proteins but also p65 and AKT phosphorylation was reduced in ISL1 knockdown cells of xenograft tumors (supplementary Fig. S2).

## Discussion

While majority of the patients with metastatic CRPC benefit from enzalutamide treatment, the responders inevitably develop resistance. Hence, studies have been directed to investigate the potential mechanisms associated with the development of enzalutamide resistance. In the present study, we demonstrate that the downregulation of ISL1 serves as an important alternative therapy for CRPC treatment through the targeting of EMT via the negative regulation of the AKT/NF-κB signaling pathway.

ISL1 serves a major role in multiple tissue types, such as heart, kidneys, skeletal muscle, nervous system, and endocrine organs, and its upregulation is associated with cancer progression and poor prognosis^14–17^. Furthermore, ISL1 can influence the expression of genes related with EMT such as ZEB1 and N-cadherin^15^. We found that ISL1 was the most highly expressed EMT factor in enzalutamide-resistant cells (Fig. 4a). We analyzed the function of ISL1 in AR signaling in PCa cells and found that cell proliferation decreased and AR signaling was downregulated in ISL1 siRNA-expressing hormone-sensitive PCa cells (Fig. 5).

As epithelial plasticity driver, Snail (a master EMT-TF) is also known to promote the development of resistance against enzalutamide through the regulation of AR activity in PCa^27^. The loss of epithelial phenotypes, including spindle morphology and intercellular adhesion, and the acquisition of mesenchymal characteristics such as high migration and invasion capacities and reduced cell-extracellular adhesion are the two major events observed during EMT^28^. The expression of EMT marker genes was downregulated in ISL1-knockdown cells in in C4-2B ENZR cells (Fig. 6b). Studies have demonstrated that EMT is associated with CRPC^29,30^. Sun et al.^31^ found that castration may induce EMT, as is evident from the decreased expression of epithelial markers (including E-cadherin) and increased levels of mesenchymal markers (including N-cadherin, Slug, Zeb1, and Twist1) in human LuCaP35 PCa xenograft tumors as well as in the normal mouse prostate tissue following androgen deprivation. Similar changes have also been reported in samples from individuals undergoing ADT^31^. EMT is driven by EMT inducing transcription factors (including Snail, Slug, Zeb1, Zeb2, and Twist), some of which have been known to be involved in the development of CRPC^28^. Shiota et al.^32^ found that castration-induced oxidative stress may promote AR overexpression through Twist1 overexpression, thereby possibly developing castration resistance. Furthermore, facilitation of castration resistance by Slug in PCa has been reported by Wu et al.^33^. Slug, another transcription factor driver of EMT, not only augments the expression of AR but also enhances AR transcriptional activity with or without androgen and acts as a novel coactivator for AR^33^. Overall, these aforementioned studies suggest that EMT is responsible for PCa progression and treatment resistance. Accordingly, treatment regimens that could reverse EMT phenotypes may become a viable alternative for CRPC therapy.

Aberrant activation of NF-κB signaling in PCa has been associated with metastatic progression^34,35^. In addition, NF-κB signaling plays an important role in EMT^36^. The knockdown of ISL1 resulted in reduced p65 phosphorylation (Fig. 6d), which is imperative for the nuclear translocation of NF-κB/p65. The NF-κB family, an important class of transcriptional regulators, comprises five members, including RelA (p65), RelB, c-Rel, p50/p105 (NF-κB1), and p52/p100 (NF-κB2). NF-κB binds to the inhibitor κB (IκB) protein in the cytoplasm in an inactive state. The IκB kinase (IKK) complex is activated under pathological conditions and subsequently induces the phosphorylation of IκB, leading to the degradation of IκB and translocation of NF-κB to the nucleus^37^. Increasing results indicate that the NF-κB transcription factor family is a crucial mediator of EMT^38^. Certain studies have shown that NF-κB binds to the promoters of genes associated with EMT, including those encoding Snail, Slug, and Twist, and increases their transcription^38,39^. Ozes et al.^40^ reported the involvement of AKT in the activation of NF-κB by mediating the phosphorylation of IKKA which is responsible for the activation of its downstream target IκB. In the current study, the knockdown of ISL1 resulted in the inhibition of the phosphorylation of both AKT and p65. These results show that ISL1 knockdown suppresses EMT by negatively regulating the AKT/NF-κB pathway (Supplementary Fig. S3). Furthermore, the AKT/NF-κB signaling pathway may drive the progression of CRPC by mechanisms other than EMT induction. Activation of NF-κB mediated by PI3K/AKT increases the expression of AR via NF-κB binding to the AR promoter^41^. CRPC, previously defined as hormone-refractory PCa, is thought to be androgen dependent^42^, indicating that targeting AR may serve as an effective strategy for CRPC treatment. The present data demonstrate that ISL1 knockdown suppressed the phosphorylation of both AKT and p65; however, whether the effect of ISL1 on the AR signaling axis occurs through the regulation of the AKT/NF-κB pathway is unclear and warrants further examination. In addition, a subsequent study is needed to determine the genomic distribution of a critical EMT regulator in CRPC using chromatin immunoprecipitation followed by next generation sequencing (ChIP-seq).

Regarding adaptive mechanisms for maintaining AR signaling in CRPC, which has been not fully elucidated, its cross-talk with other signal transduction may be involved in progression and the therapeutic resistance of PCa^43–45^. ISL1 and AR signaling were highly associated in enzalutamide resistant PCa. This raises the intriguing question of whether the therapeutic resistance of PCa depends on a complex interplay between AR signaling and ISL1. In conclusion, the knockdown of ISL1 resulted in not only inhibition of AR signaling, but also suppression of the AKT/NF-κB signaling pathway and the expression of a prominent EMT inducer. Our findings suggest that aberrant expression of ISL1 may influence enzalutamide resistance through EMT process and ISL1 seems to be a novel potential target for overcoming therapeutic resistance in the clinical setting of advanced CRPC.

## Supplementary Information

Supplementary Figures.

## References

[1] Armstrong AJ, Garrett-Mayer E, de Wit R, Tannock I, Eisenberger M (2010). Prediction of survival following first-line chemotherapy in men with castration-resistant metastatic prostate cancer. Clin. Cancer Res..

[2] Donkena KV, Yuan H, Young CY (2010). Recent advances in understanding hormonal therapy resistant prostate cancer. Curr. Cancer Drug Targets.

[3] Ferraldeschi R, Welti J, Luo J, Attard G, de Bono JS (2015). Targeting the androgen receptor pathway in castration-resistant prostate cancer: Progresses and prospects. Oncogene.

[4] Rodriguez-Vida A, Galazi M, Rudman S, Chowdhury S, Sternberg CN (2015). Enzalutamide for the treatment of metastatic castration-resistant prostate cancer. Drug Des. Dev. Ther..

[5] Liu C (2015). Intracrine androgens and AKR1C3 activation confer resistance to enzalutamide in prostate cancer. Cancer Res..

[6] Li Y (2013). Androgen receptor splice variants mediate enzalutamide resistance in castration-resistant prostate cancer cell lines. Cancer Res..

[7] Kong Y (2018). Inhibition of cholesterol biosynthesis overcomes enzalutamide resistance in castration-resistant prostate cancer (CRPC). J. Biol. Chem..

[8] Zhang Z (2018). Inhibition of the Wnt/beta-catenin pathway overcomes resistance to enzalutamide in castration-resistant prostate cancer. Cancer Res..

[9] Scher HI, Sawyers CL (2005). Biology of progressive, castration-resistant prostate cancer: Directed therapies targeting the androgen-receptor signaling axis. J. Clin. Oncol..

[10] Yilmaz M, Christofori G (2009). EMT, the cytoskeleton, and cancer cell invasion. Cancer Metastasis Rev..

[11] Thiery JP, Acloque H, Huang RY, Nieto MA (2009). Epithelial–mesenchymal transitions in development and disease. Cell.

[12] Lyttle BM (2008). Transcription factor expression in the developing human fetal endocrine pancreas. Diabetologia.

[13] Cai CL (2003). Isl1 identifies a cardiac progenitor population that proliferates prior to differentiation and contributes a majority of cells to the heart. Dev. Cell.

[14] Pfaff SL, Mendelsohn M, Stewart CL, Edlund T, Jessell TM (1996). Requirement for LIM homeobox gene Isl1 in motor neuron generation reveals a motor neuron-dependent step in interneuron differentiation. Cell.

[15] Guo T (2019). ISL1 predicts poor outcomes for patients with gastric cancer and drives tumor progression through binding to the ZEB1 promoter together with SETD7. Cell Death Dis..

[16] Li L, Sun F, Chen X, Zhang M (2018). ISL1 is upregulated in breast cancer and promotes cell proliferation, invasion, and angiogenesis. Onco Targets Ther..

[17] Shi Q (2016). ISL1, a novel regulator of CCNB1, CCNB2 and c-MYC genes, promotes gastric cancer cell proliferation and tumor growth. Oncotarget.

[18] Guo T (2011). ISL1 promotes pancreatic islet cell proliferation. PLoS One.

[19] Brønnum H (2013). Islet-1 is a dual regulator of fibrogenic epithelial-to-mesenchymal transition in epicardial mesothelial cells. Exp. Cell Res..

[20] Jarvinen PM, Laiho M (2012). LIM-domain proteins in transforming growth factor beta-induced epithelial-to-mesenchymal transition and myofibroblast differentiation. Cell. Signal..

[21] Shibue T, Weinberg RA (2017). EMT, CSCs, and drug resistance: The mechanistic link and clinical implications. Nat. Rev. Clin. Oncol..

[22] Albert M, Helin K (2010). Histone methyltransferases in cancer. Semin. Cell Dev. Biol..

[23] Langmead B, Salzberg SL (2012). Fast gapped-read alignment with Bowtie 2. Nat. Methods.

[24] Gentleman RC (2004). Bioconductor: Open software development for computational biology and bioinformatics. Genome Biol..

[25] Haile S, Sadar MD (2011). Androgen receptor and its splice variants in prostate cancer. Cell. Mol. Life Sci..

[26] Wadosky KM, Koochekpour S (2017). Androgen receptor splice variants and prostate cancer: From bench to bedside. Oncotarget.

[27] Ware KE (2016). Snail promotes resistance to enzalutamide through regulation of androgen receptor activity in prostate cancer. Oncotarget.

[28] Lamouille S, Xu J, Derynck R (2014). Molecular mechanisms of epithelial–mesenchymal transition. Nat. Rev. Mol. Cell Biol..

[29] Nakazawa M, Kyprianou N (2017). Epithelial–mesenchymal-transition regulators in prostate cancer: Androgens and beyond. J. Steroid Biochem. Mol. Biol..

[30] Li P, Yang R, Gao WQ (2014). Contributions of epithelial–mesenchymal transition and cancer stem cells to the development of castration resistance of prostate cancer. Mol. Cancer.

[31] Sun Y (2012). Androgen deprivation causes epithelial–mesenchymal transition in the prostate: Implications for androgen-deprivation therapy. Cancer Res..

[32] Shiota M (2010). Castration resistance of prostate cancer cells caused by castration-induced oxidative stress through Twist1 and androgen receptor overexpression. Oncogene.

[33] Wu K (2012). Slug, a unique androgen-regulated transcription factor, coordinates androgen receptor to facilitate castration resistance in prostate cancer. Mol. Endocrinol..

[34] Lessard L (2006). Nuclear localization of nuclear factor-kappaB p65 in primary prostate tumors is highly predictive of pelvic lymph node metastases. Clin. Cancer Res..

[35] Ismail HA, Lessard L, Mes-Masson AM, Saad F (2004). Expression of NF-kappaB in prostate cancer lymph node metastases. Prostate.

[36] Min C, Eddy SF, Sherr DH, Sonenshein GE (2008). NF-kappaB and epithelial to mesenchymal transition of cancer. J. Cell. Biochem..

[37] Chaffer CL (2013). Poised chromatin at the ZEB1 promoter enables breast cancer cell plasticity and enhances tumorigenicity. Cell.

[38] Spaderna S (2006). A transient, EMT-linked loss of basement membranes indicates metastasis and poor survival in colorectal cancer. Gastroenterology.

[39] Graham TR (2008). Insulin-like growth factor-I-dependent up-regulation of ZEB1 drives epithelial-to-mesenchymal transition in human prostate cancer cells. Cancer Res..

[40] Okugawa Y (2012). Clinical significance of Zinc finger E-box Binding homeobox 1 (ZEB1) in human gastric cancer. J. Surg. Oncol..

[41] Lee SO, Lou W, Nadiminty N, Lin X, Gao AC (2005). Requirement for NF-(kappa)B in interleukin-4-induced androgen receptor activation in prostate cancer cells. Prostate.

[42] Penning TM (2015). Mechanisms of drug resistance that target the androgen axis in castration resistant prostate cancer (CRPC). J. Steroid Biochem. Mol. Biol..

[43] Carver BS (2011). Reciprocal feedback regulation of PI3K and androgen receptor signaling in PTEN-deficient prostate cancer. Cancer Cell.

[44] Wu JD (2006). Interaction of IGF signaling and the androgen receptor in prostate cancer progression. J. Cell. Biochem..

[45] Signoretti S (2000). Her-2-neu expression and progression toward androgen independence in human prostate cancer. J. Natl. Cancer Inst..

