# Supplementary material for: ISL1 promotes enzalutamide resistance in castration-resistant prostate cancer (CRPC) through epithelial to mesenchymal transition (EMT)
Source: Sci Rep. 2021 Nov 9;11:21984. doi: 10.1038/s41598-021-01003-0 (PMC8578390; doi:10.1038/s41598-021-01003-0)

**ISL1 promotes enzalutamide resistance in castration-resistant prostate cancer (CRPC) through epithelial to mesenchymal transition (EMT)**

**Authors**

Jae Duck Choi^1^, Tae Jin Kim^2^, Byong Chang Jeong^3^, Hwang Gyun Jeon^3^, Seong Soo Jeon^3^, Min Yong Kang^3^, Seon Yong Yeom^3^, Seong Il Seo^3*^

**Affiliations**

1. Department of Urology, Nowon Eulji Medical Center, Eulji University School of Medicine, Seoul, Republic of Korea
2. Division of Immunology, Department of Molecular Cell Biology and Samsung Biomedical Research Institute, Sungkyunkwan University School of Medicine, Suwon, Republic of Korea
3. Department of Urology, Samsung Medical Center, Sungkyunkwan University School of Medicine, Seoul, Republic of Korea

**Correspondence**

*Seong Il Seo MD., PhD, Department of Urology, Samsung Medical Center, Sungkyunkwan University School of Medicine, Seoul, Republic of Korea, E-mail: siseo@skku.edu


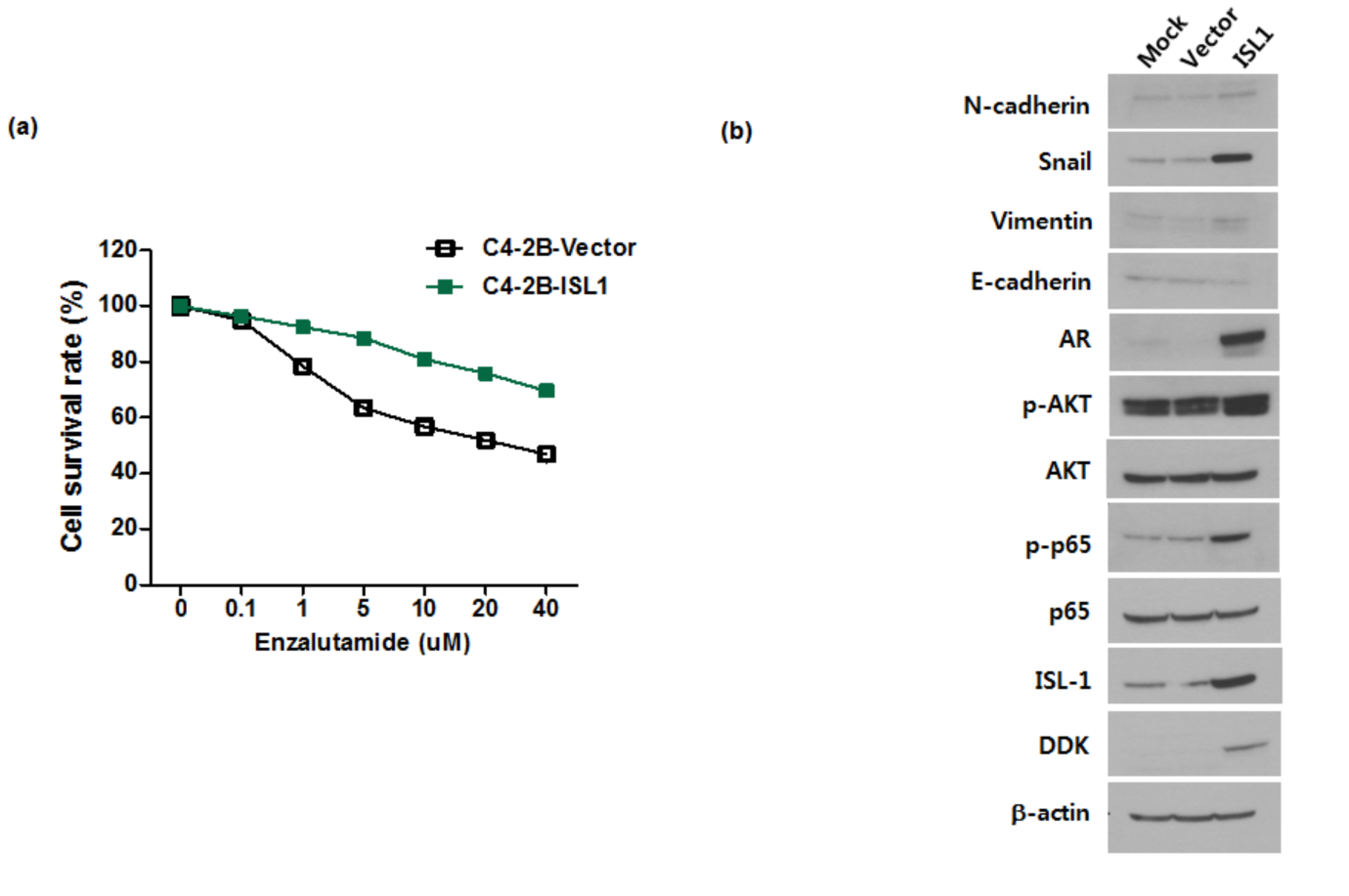


**Supplementary Figure S1: ISL1 overexpression promoted cell proliferation and EMT**. (a) Overexpression of ISL1 enhances the survival of cells treated with increasing doses of enzalutamide over 72 hours in C42B cells. The CCK-8 assay was performed to assess the proliferation ability. (b) Western blotting analysis of AR and EMT-associated proteins was shown in ISL1 overexpression of C4-2B cells. Full-length images are presented in Supplementary Fig. S9.


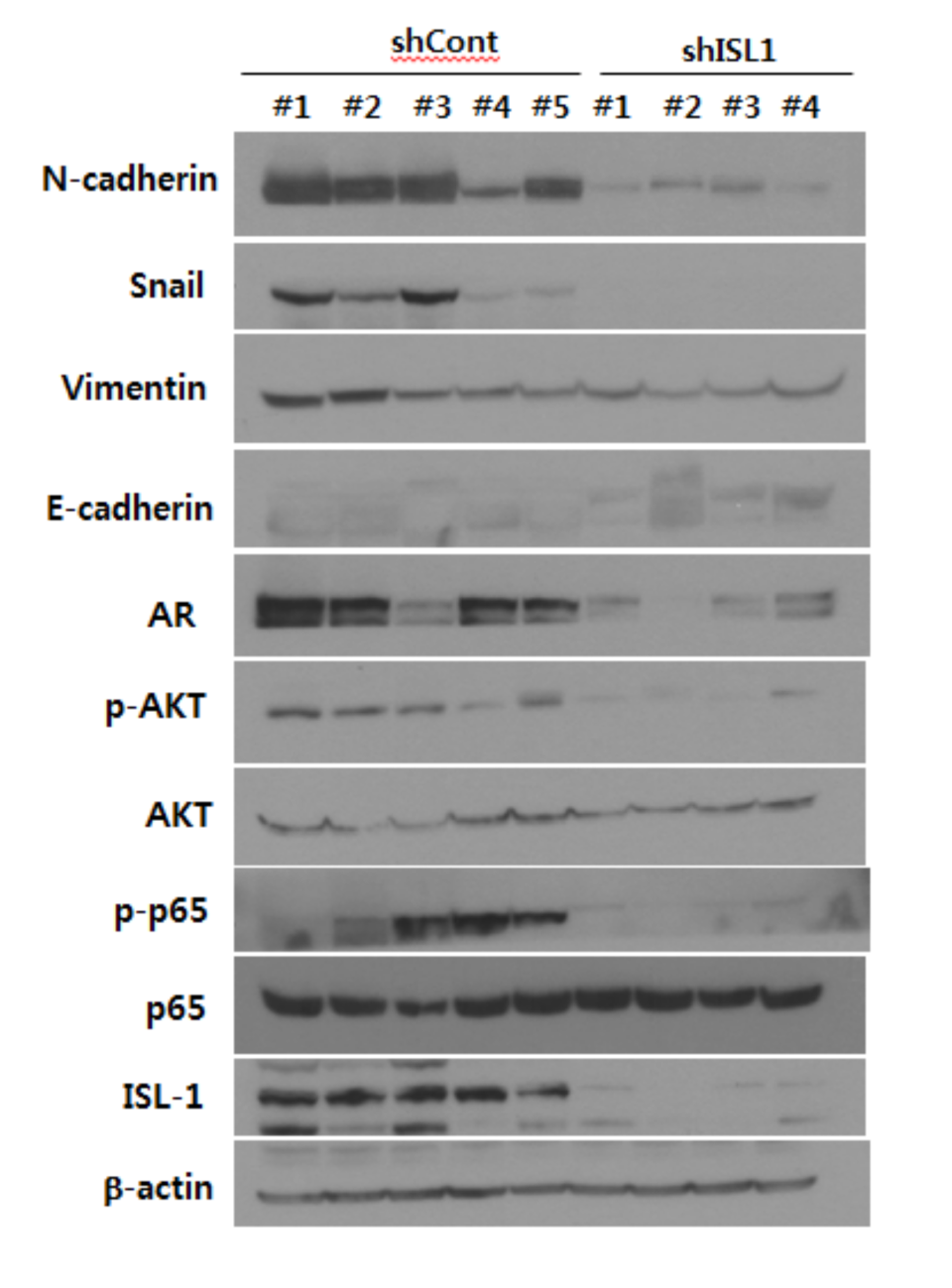


**Supplementary Figure S2: ISL1 knockdown attenuates the expression of AR and EMT-associated proteins in enzalutamide-resistant (ENZR) prostate cancer cells.** Total protein was extracted from each xenograft tumors and western blotting was carried out with the indicated antibodies. Protein levels were normalized to b-actin levels. Full-length images are presented in Supplementary Fig. S10.


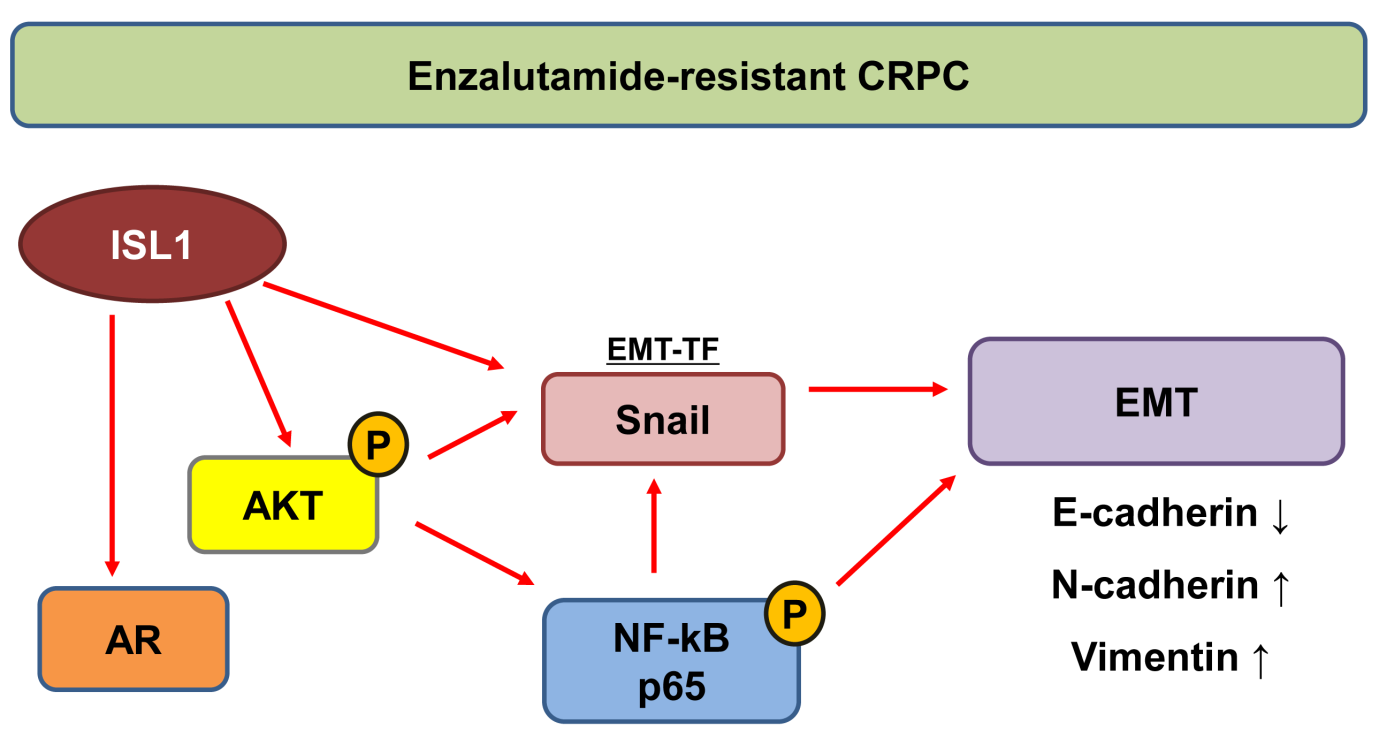


**Supplementary Figure S3: Schematic representation of the promoting effect of ISL1 on inducing EMT in enzalutamide resistant CRPC.** In response to EMT‐triggering events, such as the activation of AKT/NF-κB signaling pathways (e.g., AKT, p65, NF-Κb) or the expression of EMT‐TF (e.g., Snail), cancer cells transition from an epithelial phenotype to a mesenchymal phenotype, with the suppression of epithelial markers and expression of mesenchymal markers. As these pathways are interlinked with each other, our data indicate that upregulation of ISL1, induction of Snail, and activation of the AKT/NF-κB pathway eventually result in reactivation of AR signaling and the EMT process in red lines.

**Supplementary Figure S4: Uncropped images Figure 1**

Figure 1b-parental-0


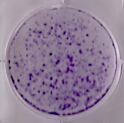


Figure 1b-parental-10


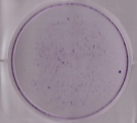


Figure 1b-parental-20


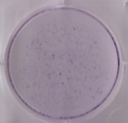


Figure 1b-ENZR-0


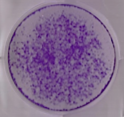


Figure 1b-ENZR-10


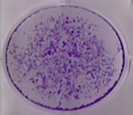


Figure 1b-ENZR-10


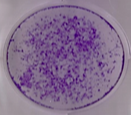


Figure 1c-parental-0 Figure 1c-parental-10


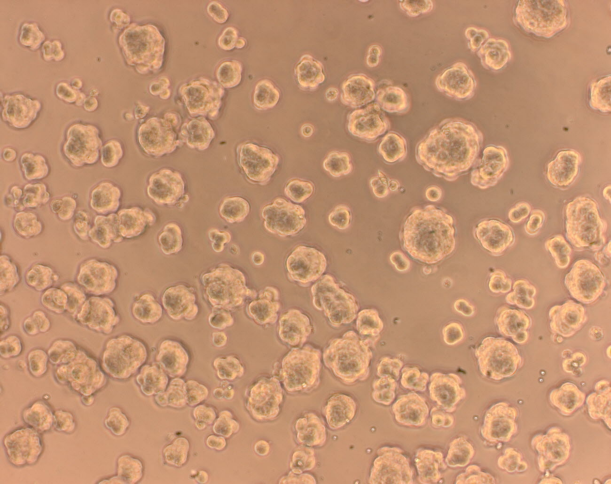

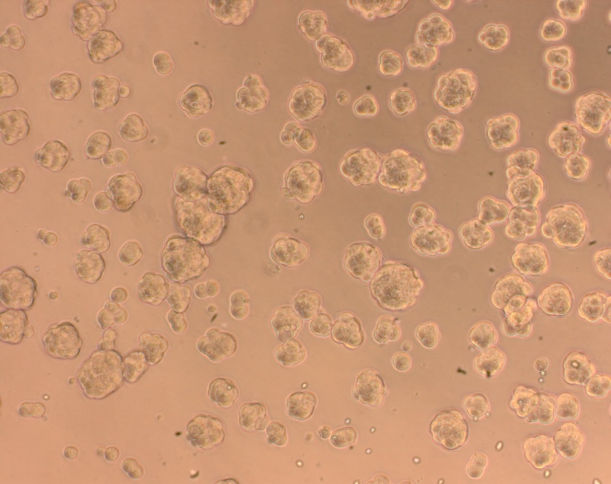


Figure 1c-parental-20 Figure 1c-ENZR-0


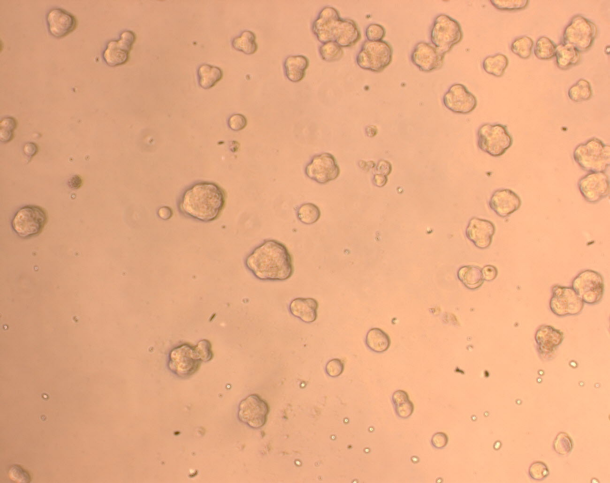

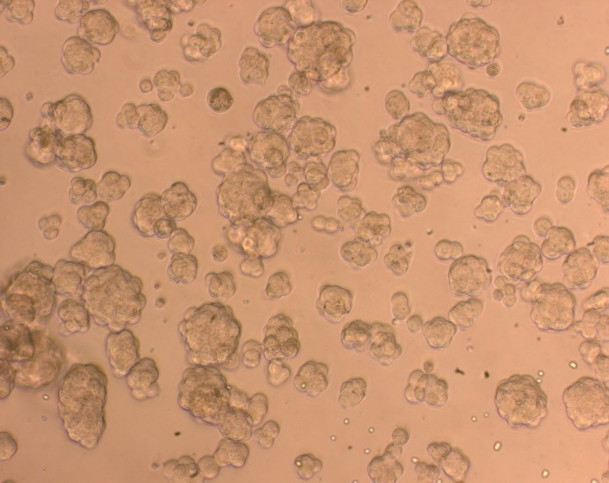


Figure 1c-ENZR-10 Figure 1c-ENZR-20


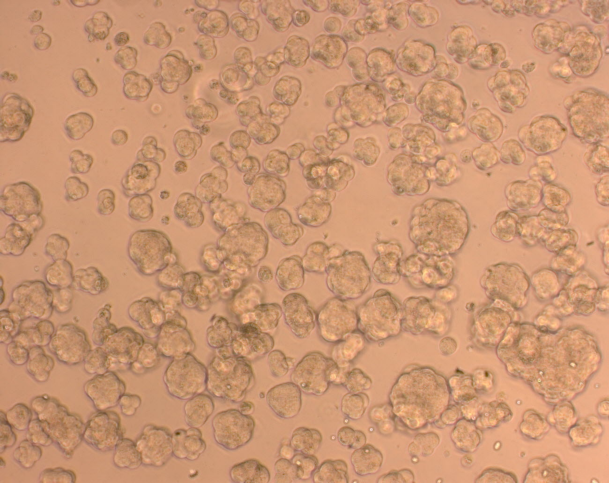

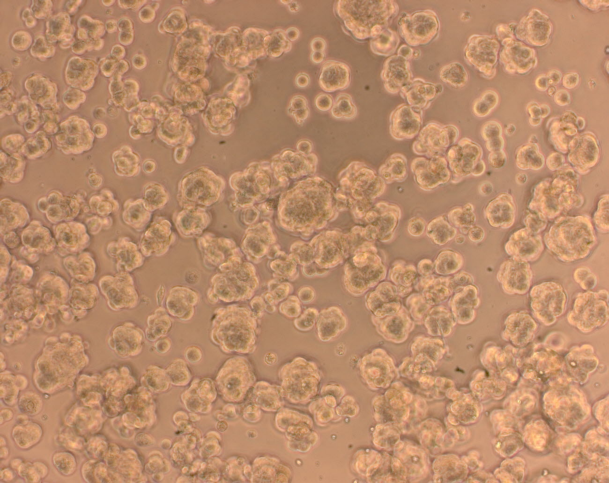


**Supplementary Figure S5: Uncropped images Figure 2**

Figure 2a, Figure 2b


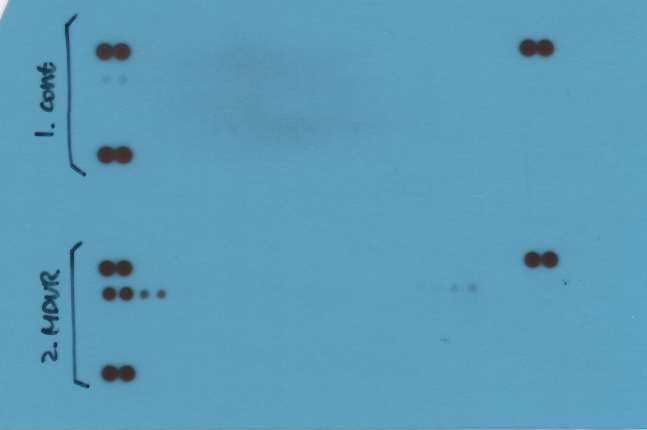


**Supplementary Figure S6: Uncropped images Figure 5**

Figure 5b


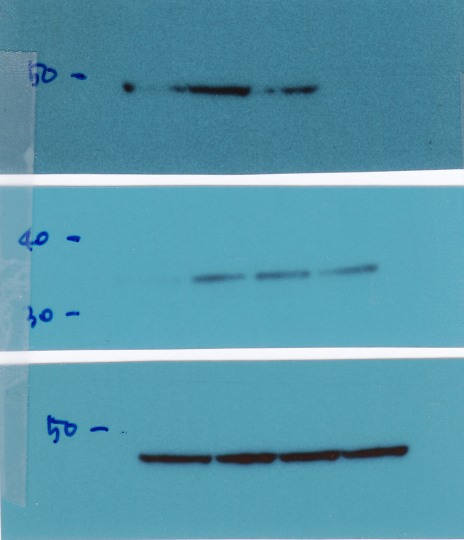


Figure 5e


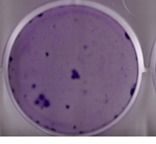
 siCont


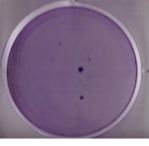
siISL1

**Supplementary Figure S7: Uncropped images Figure 6**

Figure 6a Figure 6b


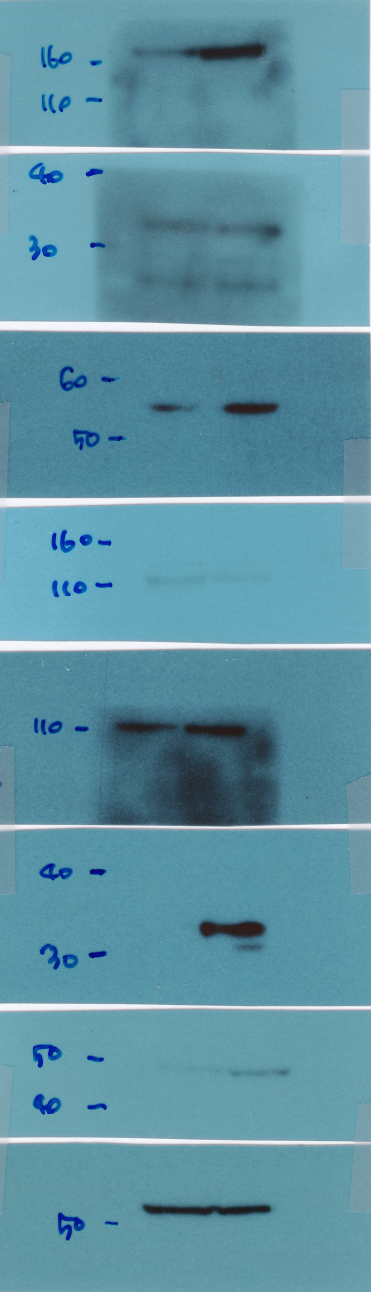

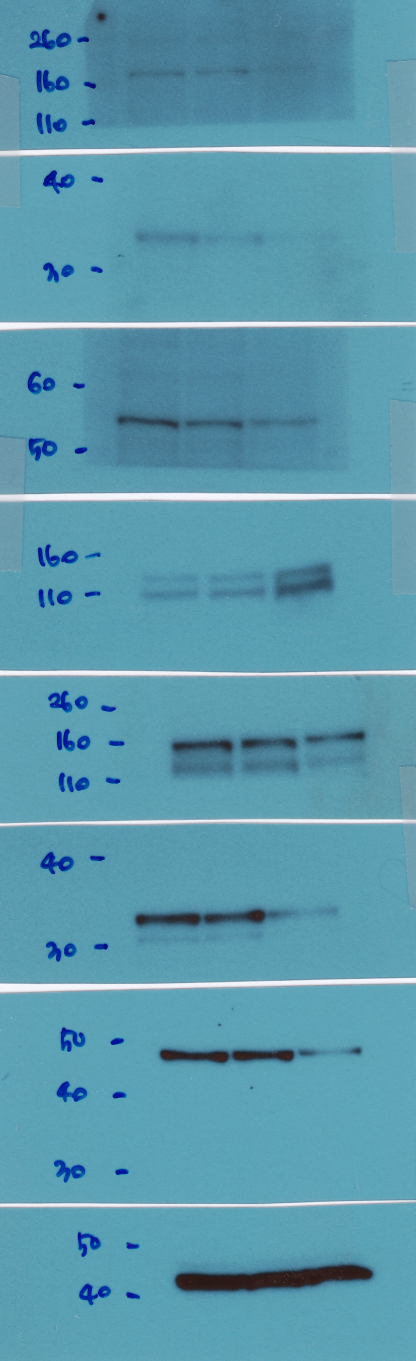


Figure 6c


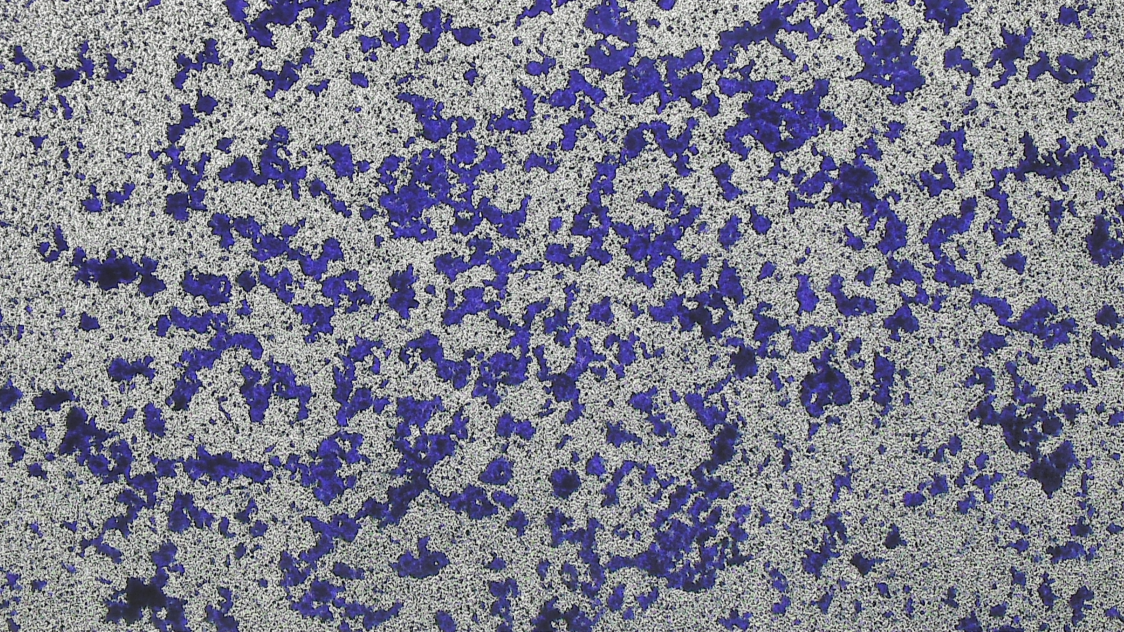
 siCont


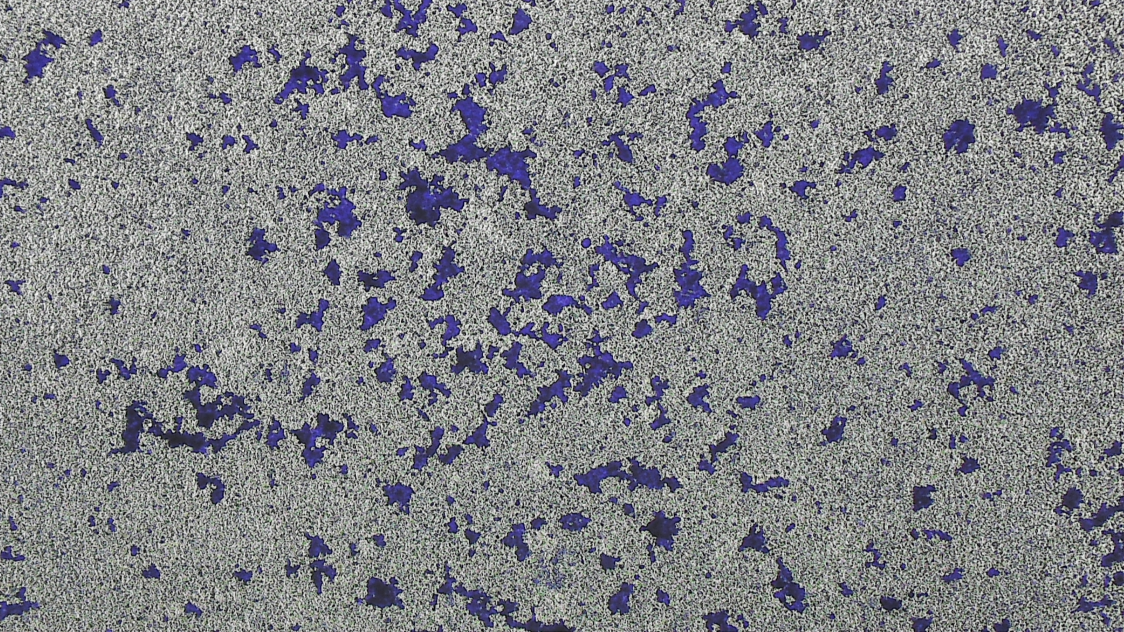
 siISL1(10 nM)


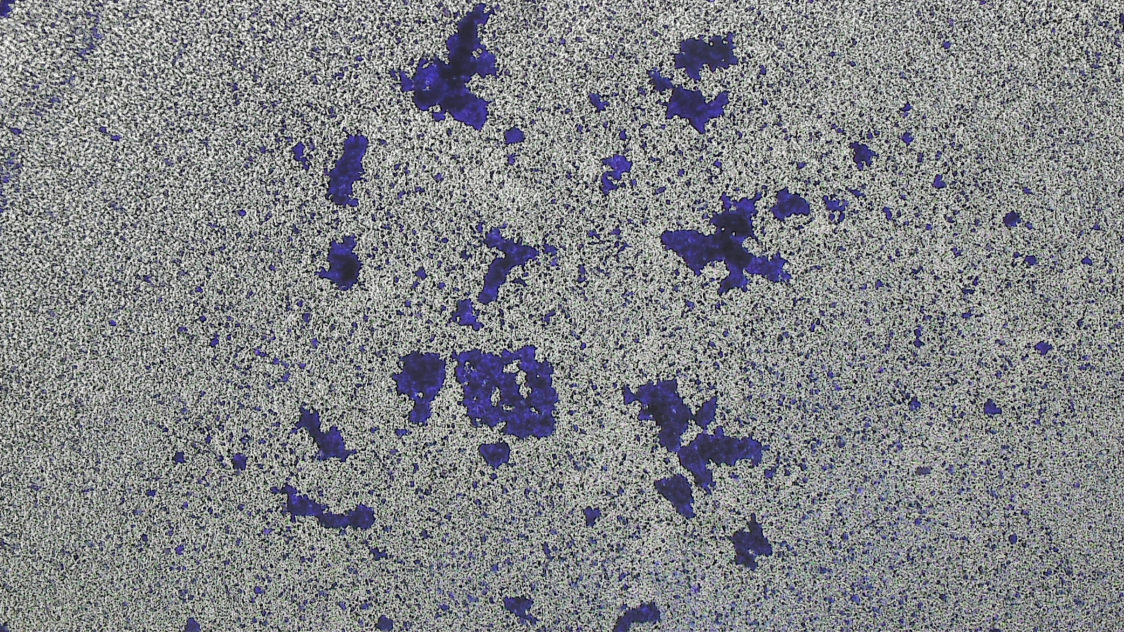
 siISL1 (30 nM)

Figure 6d


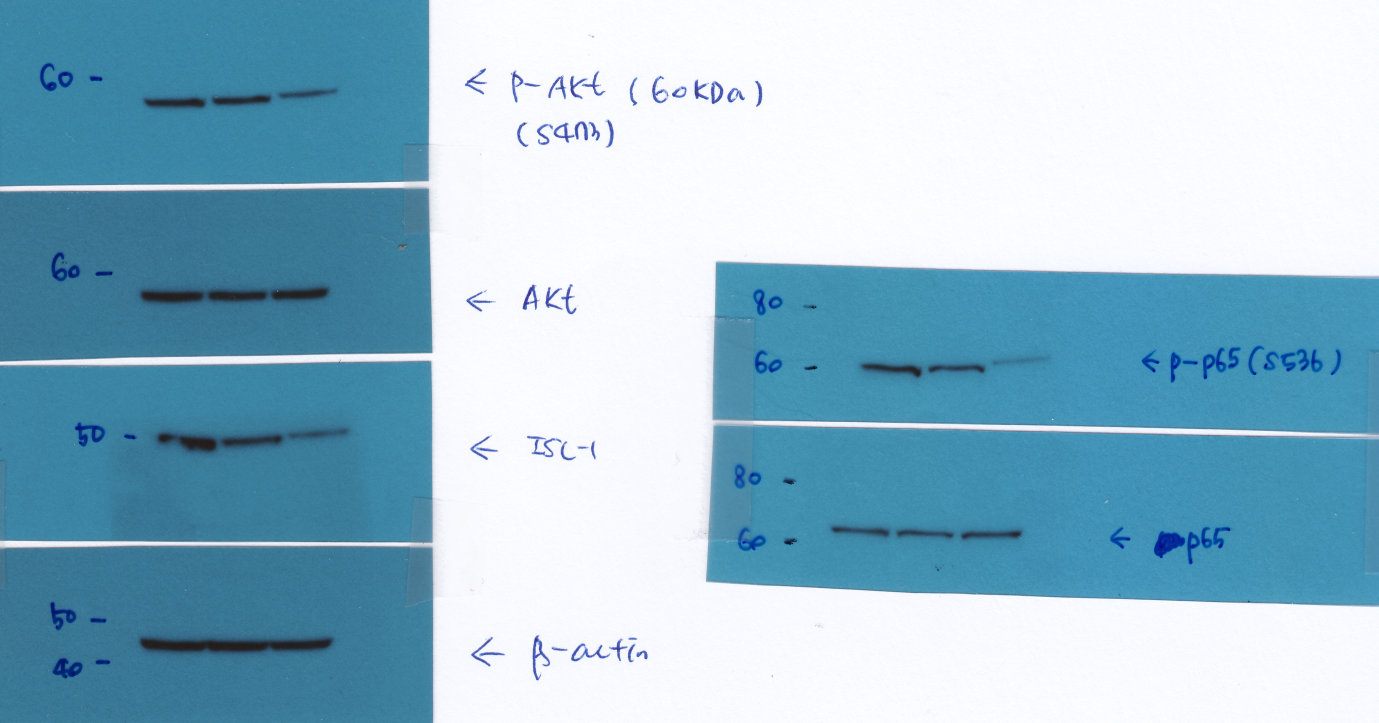


**Supplementary Figure S8: Uncropped images Figure 7**

Figure 7b


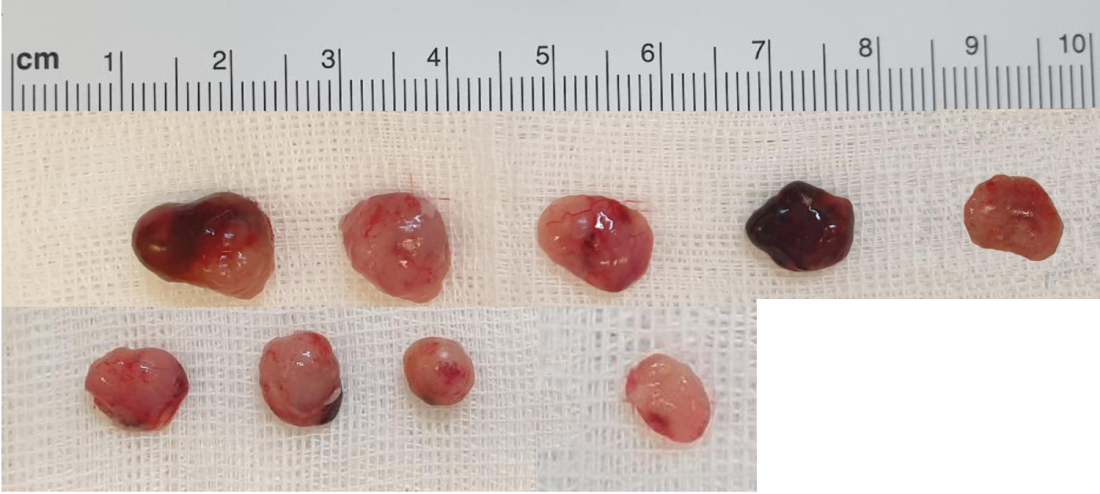


**Supplementary Figure S9: Uncropped images Supplementary Figure S1**


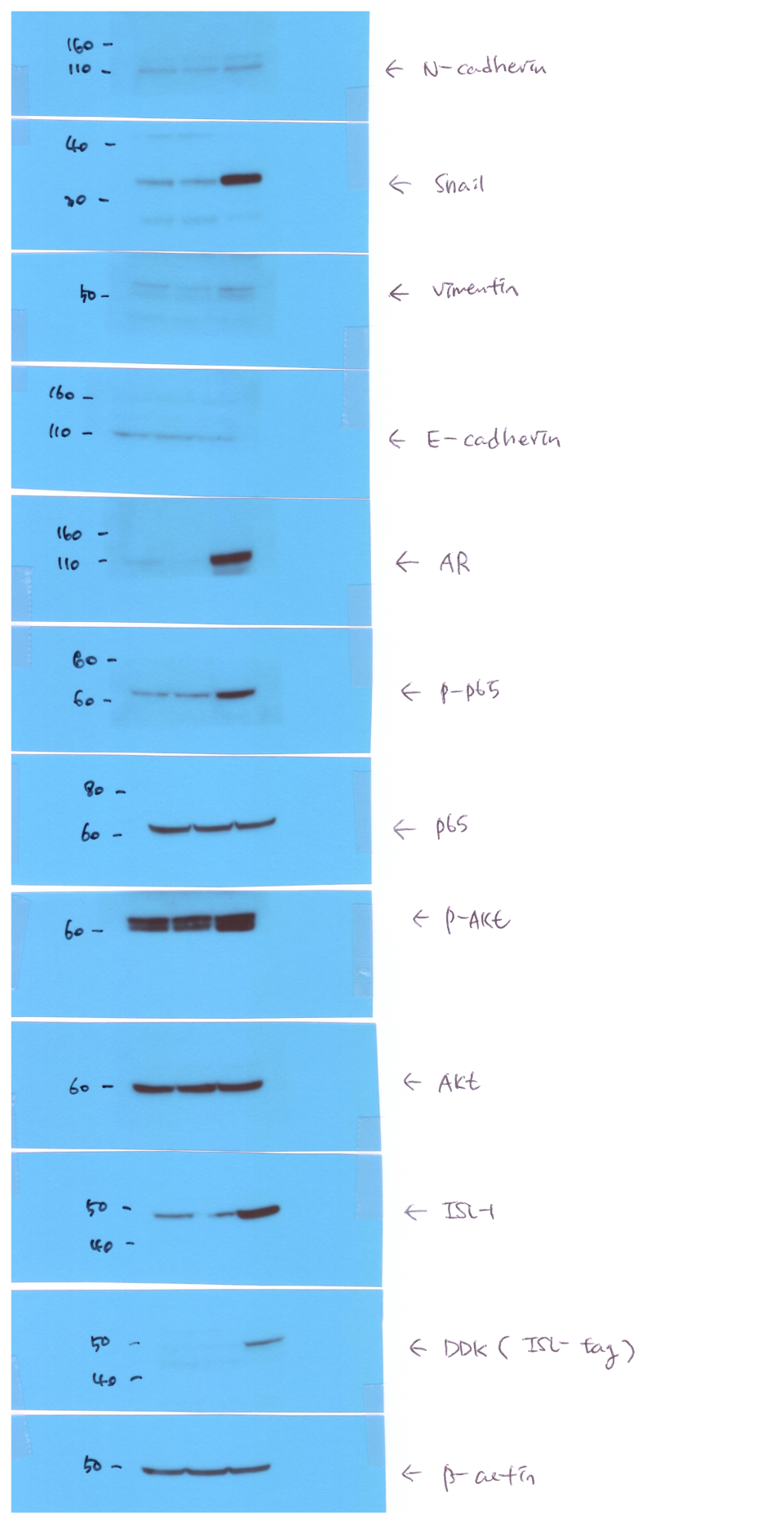


**Supplementary Figure S10: Uncropped images Supplementary Figure S2**


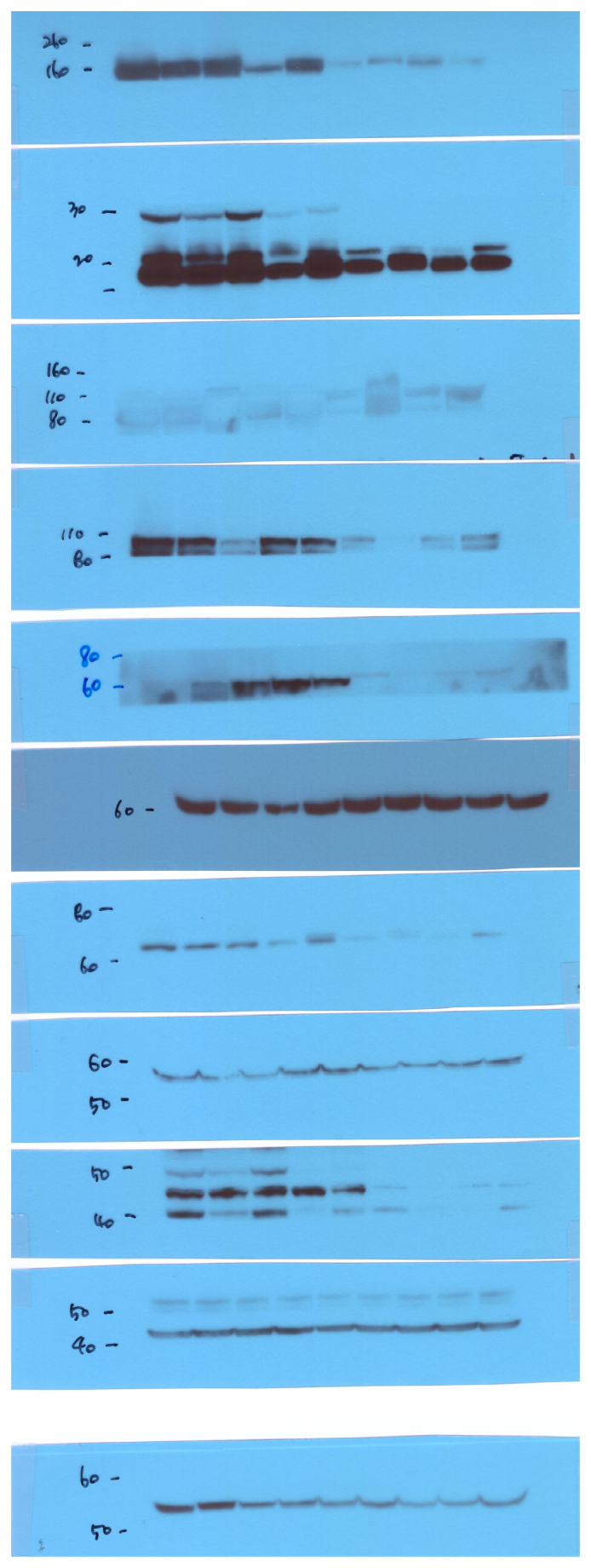

Supplement: Supplementary file 1 — Supplementary Figures. [file 41598_2021_1003_MOESM1_ESM.docx]
